# Outbreak of Novel Coronavirus (SARS-CoV-2): First Evidences From International Scientific Literature and Pending Questions

**DOI:** 10.3390/healthcare8010051

**Published:** 2020-02-27

**Authors:** Emanuele Amodio, Francesco Vitale, Livia Cimino, Alessandra Casuccio, Fabio Tramuto

**Affiliations:** Department of Health Promotion, Maternal and Infant Care, Internal Medicine and Medical Specialties, “G. D’Alessandro”, University of Palermo, Via del Vespro 133, 90127 Palermo, Italy; francesco.vitale@unipa.it (F.V.); livia.cimino@unipa.it (L.C.); alessandra.casuccio@unipa.it (A.C.); fabio.tramuto@unipa.it (F.T.)

**Keywords:** SARS-CoV-2, outbreak, COVID-19, public health

On 31 December, 2019, a cluster of 27 pneumonia cases of unknown etiology was reported by Chinese health authorities in Wuhan City (China). In particular, for almost all cases, an exposition to the Wuhan’s Huanan Seafood Wholesale Market was found and, thus, the market was considered the most probable source of the virus outbreak [1]. Chinese health authorities have taken prompt public health measures, including intensive surveillance, epidemiological investigations, and closure of the market on 1 January, 2020 (Figure 1).

On 9 January, 2020, the Chinese Government reported that the cause of the outbreak was a novel coronavirus, recently named SARS-CoV-2 (severe acute respiratory syndrome coronavirus 2) [2], and was responsible for a disease defined COVID-19 (novel coronavirus disease 2019). This virus has been detected as the causative agent for 15 of the 59 pneumonia cases [3]. 

From that date, an increasing number of studies have been published and several international institutions (World Health Organization, Centers for Disease Control and Prevention, European Centers for Disease Control and Prevention) have provided findings supporting a rapid increase in the general knowledge. However, despite these significant improved data, many questions about the new coronavirus remain, and answers could be strategic for programming and designing public health interventions.

SARS-CoV-2 was found to be a β-Coronavirus of group 2B with at least 70% similarity in genetic sequence to SARS-CoV-1, but sufficiently divergent to be considered a new human-infecting betacoronavirus (Table 1) [4]. It is highly probable that genome differences between SARS-CoV-1 and SARS-CoV-2 could be responsible for the different functionality and pathogenesis; thus, further studies could significantly help to solve this gap. The genetic sequence of the SARS-CoV-2 has been shared on 10 January, 2020, in order to allow the production of specific diagnostic PCR tests in different countries for detecting the novel infection [5]. 

The evident convergence between SARS-CoV-2 and bat coronavirus (at least 96% identical at the whole-genome level) seems to suggest that bats could be the original host [6]. A possible role of civets, snakes, and pangolins is not excluded as potential intermediate hosts, and it is clear that tracking the path of the virus could be crucial for preventing further exposure and outbreaks in the future.

The SARS-CoV-2 RNA sequences have been found to have limited variability and the estimated mutation rates in coronavirus, which SARS-CoV-2 phylogenetically links to, are moderate to high, compared to the others in the category of single-stranded RNA viruses [7]. However, an accurate measure of the mutation rate for SARS-CoV-2 has not been calculated and the evaluation of its genetic evolution over time could have important implications for strategic planning in the prevention, as well as in the development of vaccines and antibodies-based therapies.

Another important key point is the role of humoral immunity that, as for other coronavirus, might not be strong or long-lasting enough to keep patients safe from contracting the disease again.

After infection occurred, incubation has been estimated to vary from 5 to 6 days, with a range of up to 14 days [8]. However, the knowledge of the true incubation time could improve the estimates of the rates of asymptomatic and subclinical infections among immunocompetent individuals; thus, increasing the specificity in detecting COVID-19 cases. Additionally, it could significantly change the forecasting projection models on the worldwide outbreak evolution. 

In this sense, recently published studies have estimated a basic reproductive number of 3.28, exceeding the initial World Health Organization (WHO) estimates of 1.4 to 2.5 [9]. The basic reproductive number is an indication of viral transmissibility, representing the average number of new infections generated by a single infectious person in a totally naïve population; thus, when it decreases below 1, the outbreak can be considered under control. Moreover, there are evidences that SARS-CoV-2 appears to have been transmitted during the incubation period of patients in whom the illness was brief and nonspecific, whereas the detection of SARS-CoV-2 with a high viral load in the sputum of convalescent patients arouse concern about prolonged shedding of the virus after recovery [10]. 

In symptomatic COVID-19 patients, illness may evolve over the course of a week or longer, beginning with mild symptoms that progress (in some cases) to the point of dyspnea and shock [11]. Most common complaints are fever (almost universal), cough, which may or may not be productive, whereas myalgia and fatigue are relatively common conditions [12].

The updated case fatality rate of diagnosed cases is 2.3%, with an increasing risk in subjects aged 60 and older (3.6% in subjects 60–69 years old; 8% in subjects 70–79 years old; and 14.8% in subjects aged 80 and older), and those with comorbidities (case fatality rate in healthy subjects was 0.9%) [13]. Moreover, fatality rates seem to be decreasing over time (15.6%, 1–10 January, 2020; 5.7%, 11–20 January, 2020; 1.9%, 21–31 January, 2020; 0.8% after 1 February, 2020) although this finding could be due to the increasing detection of “mild” cases in the general population or to a better management of the disease [14].

Unfortunately, to date, there are no vaccines against SARS-CoV-2, and there is the awareness that several months may be required to undergo extensive testing, and determine vaccine safety and efficacy before a potential wide use. Similarly, there is no single specific antiviral therapy; COVID-19 and the main treatments are supportive care (e.g., supportive therapy and monitoring—oxygen therapy and fluid management). In the last days, recombinant interferon (IFN) with ribavirin and infusions of blood plasma from people who have recovered from the COVID-19 are under evaluation, to treat infected subjects with encouraging results [14].

In conclusion, it is evident that in just a few weeks, the international scientific community has been involved in producing well-documented evidences in order to increase general knowledge about epidemiology, immunopathology, prevention, and treatment of COVID-19. However, many doubts about the new coronavirus remain, whereas there is the conviction that finding and sharing answers to these questions could represent a major challenge for public health control of a possible global SARS-CoV-2 outbreak. 

## Figures and Tables

**Figure 1:**
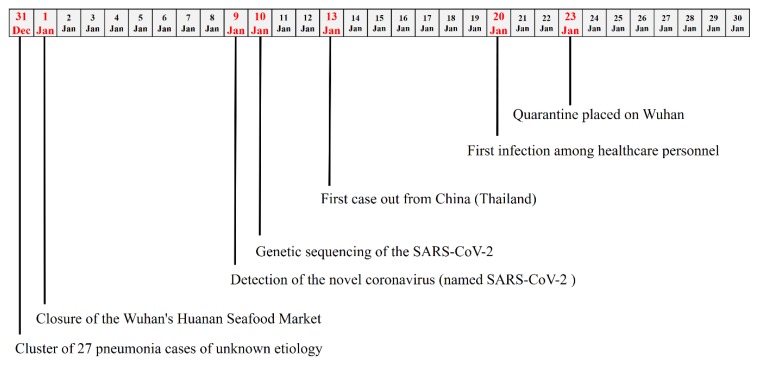
Timeline of the key events observed in the first month of the 2019 severe acute respiratory syndrome coronavirus 2 (SARS-CoV-2) outbreak.

**Table 1:** Summary of the scientific evidences, suggestions, and pending questions on the 2019 SARS-CoV-2 outbreak.

	Documented Evidences	Scientific Suggestions	Pending Questions
Causative Agent	Family of Coronaviridae (genus: Betacoronavirus) enveloped positive single-stranded RNA [4,15].		
Virus emergence	31 December, 2019 [1].	Emergence of SARS-CoV-2 into the human population likely occurred in mid-November 2019 [16].	
Virus phylogenesis	SARS-CoV-2 is genetically very close to SARS-CoV, but sufficiently divergent to be considered a new human-infecting betacoronavirus [4].		Genome differences between SARS-CoV and SARS-CoV-2 could be responsible for the different functionality and pathogenesis.
Virus hosts	SARS-CoV-2 is 96% identical at the whole-genome level to a bat coronavirus [4].		Cat civets, snakes, and pangolins are indicated as potential intermediate hosts. Tracking the path of the virus could be crucial for preventing further exposure and outbreaks in the future.
Virus mutation rate and adaptation		The SARS-CoV-2 RNA sequences have limited variability and the estimated mutation rates in coronavirus, which SARS-CoV-2 phylogenetically links to, are moderate to high compared to the others in the category of ssRNA viruses [7]. In healthcare workers, severity of disease showed a decreasing trend over time (critical or severe presentation was observed in 45%, 1–10 January, 2020; 19.7%, 11–20 January, 2020; 14.4%, 21–31 January, 2020; 8.7% after 1 February, 2020) [13].	The outbreak could be initiated from either a single introduction into humans or from very few animal-to-human transmission events. How SARS-CoV-2 evolves over time could have important implications for both strategic planning in public health interventions, prevention of, and development of vaccines and antibodies.
Virus environmental persistence		On inanimate surfaces, human coronaviruses can remain infectious for up to 9 days. A surface disinfection with 0.1% sodium hypochlorite, 0.5% hydrogen peroxide, or 62%–71% ethanol can be regarded as effective against coronaviruses within 1 min [15,17].	
Virus spreading to human	SARS-CoV-2 spreads from person-to-person via respiratory droplets (coughs or sneezes) and possibly also via contaminated hands or surfaces. Close contact (within about 6 feet) increases risk of transmission [18].	The risk of transmission seems to be proportional to the severity of patient symptoms [18].	
Immunity duration			As for other coronaviruses, in infected patients, humoral immunity might not be strong or long-lasting enough to keep them from contracting the disease again.
Incubation period	Current estimates suggest a mean incubation period of 6.4 days (95% credible interval: 5.6–7.7), with a range from 2.1 to 11.1 days (2.5th to 97.5th percentile) [19]. To date, the maximum observed incubation period was 14 days [8].	A recent modelling study confirmed that it remains prudent to consider the incubation period of at least 14 days [20].	A longer incubation time may lead to a high rate of asymptomatic and subclinical infection among immunocompetent individuals. This finding could represents a key question for setting length of surveillance period for each at risk patient.
Basic reproductive number	A basic reproductive number of 3.28 has been estimated by a review (range between different studies 1.95 to 6.49) [9].		It is important to further assess the reason for the higher basic reproductive number values estimated by some mathematical studies. Reproductive number estimations produced at later stages can be expected to be more reliable, as they build upon more case data and include the effect of awareness and intervention. Variations in reproduction number could also be found over time, according to an improving capacity to detect cases.
Duration of the disease			To date, there are no evidences on this key point. The detection of SARS-CoV-2 and a high sputum viral load in a convalescent patient arouse concern about prolonged shedding of SARS-CoV-2 after recovery [10].
Asymptomatic carriers	There are evidences that SARS-CoV-2 appears to have been transmitted during the incubation period of a patient, in whom the illness was brief and nonspecific [10].		The fact that asymptomatic persons are potential sources of SARS-CoV-2 infection may warrant a reassessment of transmission dynamics of the current outbreak.
Frequent symptoms	In symptomatic patients, illness may evolve over the course of a week or longer, beginning with mild symptoms that progress (in some cases) to the point of dyspnea and shock [11]. Most common complaints are fever (almost universal), cough, which may or may not be productive, whereas myalgia and fatigue are common [12]. About 80% of identified cases were mild cases [13].		
Severe clinical presentations	Most common complications are: (1) acute respiratory distress syndrome; (2) septic shock; (3) acute kidney injury; (4) myocardial injury; (5) secondary bacterial and fungal infections; (6) multiorgan failure [11,12]. About 14% of identified cases were severe and 4.7% critical [13].		
Case fatality rate	The updated case fatality rate of diagnosed cases is 2.3, with increasing risk for subjects aged 60 or older (3.6% in 60–69 year olds; 8% in 70–79 year olds, and 14.8% in subjects 80 or older), and in those with comorbidities (case fatality rate in healthy subjects was 0.9%) [13]. Moreover, mortality rates seem to be decreasing over time (15.6%, 1–10 January, 2020; 5.7%, 11–20 January, 2020; 1.9%, 21–31 January, 2020; 0.8% after 1 February, 2020) [13].		
Prevention in the general population	For the general public, the best way to prevent illness is to avoid being exposed to this virus. Face masks do not seem to be as effective in protecting those who are not infected, and wearing a mask could be useful only when taking care of a person with suspected COVID-19 [20].		
Vaccines	There are currently no vaccines against coronaviruses, including SARS-CoV-2.		Various vaccine strategies against coronavirus, such as using inactivated viruses, live-attenuated viruses, viral vector-based vaccines, subunit vaccines, and recombinant proteins are under evaluation. However, several months may be required to undergo extensive testing to determine its safety and efficacy and before it can be widely used [21].
Therapies	At present, there is no single specific antiviral therapy for SARS-CoV-2 and the main treatments are supportive care (e.g., supportive therapy and monitoring—oxygen therapy and fluid management). Recombinant interferon (IFN) with ribavirin only has limited effects against CoVs infection [14].		Infusions of blood plasma from people who have recovered from the COVID-19 could represent a valid approach to treat those still battling the infection.
